# Comparison of the Survival Rate Against Fracture of Endodontically Treated Premolars with Exposed Cervical Lesions Restored with Crowns and Resin Composites: A Retrospective Study

**DOI:** 10.14744/eej.2021.21939

**Published:** 2022-03-07

**Authors:** Kantaporn KAEWCHOMPHOO, Danuchit BANOMYONG, Yaowaluk NGOENWIWATKUL, Piyapanna PUMPALUK

**Affiliations:** From the Department of Operative Dentistry and Endodontics (K.K., D.B. 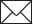 danuchit.ban@mahidol.ac.th), Mahidol University Faculty of Dentistry, Bangkok, Thailand; Department of Community Dentistry (Y.N.), Mahidol University Faculty of Dentistry, Bangkok, Thailand; Department of Advanced General Dentistry (P.P.), Mahidol University Faculty of Dentistry, Bangkok, Thailand

**Keywords:** Cervical caries, dental restoration, endodontically treated teeth, survival rate, tooth abrasion, tooth fractures

## Abstract

**Objective:**

The purpose of this study was to evaluate the effect of coronal restorations on the survival rates against fracture of endodontically treated premolars with exposed cervical lesions and to identify the prognostic factors for fracture.

**Methods:**

Data of the endodontically treated premolars with exposed cervical lesions restored with resin composites or crowns between 2011 and 2020 were collected. The presence of a fracture was recorded, and the possible prognostic factors were recorded. Statistical analyses were performed, with a significance level of P<0.05, using a Kaplan-Meier survival analysis, log-rank tests, and Cox proportional hazard models were used to identify the prognostic factors.

**Results:**

The survival rates against fracture were not significantly different between the teeth restored with crowns (93.3%) or resin composites (86%) (P≥0.05). A high frequency of non-restorable fractures was observed in both groups. Crestal bone reduction to the middle-third of the root was identified as the significant prognostic factor (P<0.05).

**Conclusion:**

For endodontically treated premolars with exposed cervical lesions, resin composite restorations provided a high comparable survival rate that was comparable to that of crowns. A higher risk of fracture was found in endodontically treated premolars with crestal bone loss to the middle-third of the root.

**HIGHLIGHTS**

• This is the first clinical study to assess the effect of coronal restorations (crown or resin composite) on the survival against fracture of endodontically treated teeth with an exposed cervical lesion.
• Endodontically treated premolars with an exposed cervical cavity could be permanently restored with direct resin composite that extended into the root canal below the exposure site; a full crown is not typically required.
• The premolars with crestal bone loss to the middle-third of root had a higher risk of fracture. Thus, the supporting periodontal tissues should be well-maintained to prevent a decrease in the bone level that increases the risk of fracture.

## INTRODUCTION

The clinical success of endodontic treatment significantly improves when the tooth recieves a coronal restoration that prevents coronal leakage and protects the remaining tooth structure from fracture (1, 2). Endodontically treated teeth (ETT) are weakened from the marked loss of tooth structure and are, therefore, prone to fracture (3). An appropriate restorative plan should be carefully considered to protect ETT and improve their longevity (4).

Endodontically treated posterior teeth restored with full coverage crowns display a significantly lower fracture incidence than those restored with resin composites (5). However, endodontically treated premolars with adequate remaining tooth structure, i.e. the loss of only one marginal ridge, can be successfully and conservatively restored with resin composite (6, 7). In addition, the absence of parafunctional habits and the presence of two adjacent teeth that distribute the occlusal force significantly improves the survival rate against ETT fracture (7).

A cervical lesion refers to the loss of cervical tooth structure on the buccal or lingual surface due to root caries or tooth abrasion. The cervical cavity generates high stress that is concentrated at the cervical region, especially at the pulpal floor of the cavity (8-10). As the lesion depth increases, the stress in the cervical region gradually increases, while the fracture resistance significantly declines (10). However, the stress accumulation and the decrease in fracture strength are reversible after the cavity is restored with resin composite (11-13).

A cervical fracture can occur in ETT with exposed cervical lesions and due to this concern, many dentists typically choose a full-coverage crown compared with a resin composite restoration. However, this preference lacks supporting clinical evidence. In a laboratory study by Machado et al. (2017), (11) the fracture resistance of ETT restored with all-ceramic crowns and resin composites was not significantly different. Furthermore, the resistance to fracture in both groups was comparable to that of intact teeth (11). Thus, the benefit of restoring the tooth with a crown rather than resin composite to prevent tooth fracture in ETT is questionable. Currently, the restorative management of ETT with exposed cervical lesions is based on only a few laboratory studies (8, 10-13).

It remains unclear whether the type of coronal restoration, particularly crown and resin composite, affect the survival against fracture of endodontically treated premolars with exposed cervical lesions. Moreover, the prognostic factors affecting survival have not been previously reported. Therefore, the aim of this retrospective cohort study was to compare the survival rates against fracture of endodontically treated premolars with exposed cervical lesions that were restored with either a crown or resin composite. In addition, the significant prognostic factors for fracture were identified.

## MATERIALS AND METHODS

The protocol for this retrospective cohort study was approved by the Ethical Review Committee for Human Research Office of the Faculty of Dentistry and Faculty of Pharmacy, Mahidol University, Bangkok, Thailand (MU-DT/PY-IRB 2020/DT011). Informed consent was not required due to the retrospective nature of the study. Data was collected from the dental records and radiographs of premolars receiving endodontic treatment at the Endodontic Clinic, Faculty of Dentistry, Mahidol University, and the patients were recalled between January 2011 and June 2020. The post-endodontic coronal restorations, either full-coverage crowns or resin composites, were provided by undergraduate, postgraduate, or restorative dentists. The subjects were recruited according to the following criteria.

### Selection criteria and data collection

The inclusion criteria were endodontically treated premolars (1) with exposed cervical lesions, (2) complete root formation, (3) restored with direct resin composites or full-coverage crowns, (4) occluding with natural teeth or fixed dental prostheses, (5) their clinical and radiographic records were adequate, and (6) had a recall period of at least 1 year.

The exclusion criteria were the teeth with (1) endodontic or restorative procedural error(s) that compromised the strength of the tooth, (2) endodontic access through an existing crown, (3) preoperative cracks or fractures, (4) a post was removed during root canal retreatment, and (5) concurrent orthodontic treatment.

The following data were recorded: the sex, age, tooth location, location and height of the exposed cervical lesion, type of coronal restoration, functioned as an abutment for a prosthesis, an opposing tooth, posterior tooth support, proximal contact(s), level of bone support, and parafunctional habits. In addition, the presence of a fracture was determined, and the fracture location and the restorability were recorded.

### Treatment procedures

For the endodontic treatment, conservative access openings were performed under dental dam isolation. The root canal preparation was performed with stainless steel hand files (Dentsply Maillefer, Tulsa, OK, USA) or rotary nickel-titanium files (ProTaper NEXT or WaveOne Gold; Dentsply Sirona, York, PA, USA) and irrigated with 2.5% sodium hypochlorite and 17% EDTA. A water-based calcium hydroxide intracanal medicament was used for at least 1 to 2 weeks. The obturation technique was either lateral compaction (LC) or warm vertical compaction (WVC), based on the preference of the treatment providers, using gutta-percha and either a zinc oxide eugenol root canal sealer, MU Sealer (MU Dent, Bangkok, Thailand) or an epoxy resin sealer, AH Plus (Dentsply). The gutta-percha was seared off and plugged into the canals ~1-3 mm below the cervical exposure site. The coronal access was filled with a temporary restoration.

A post-endodontic coronal restoration, either direct resin composite or crown, was planned. For the premolars restored with resin composite as a permanent restoration, a 1-2 mm thick glass-ionomer cement (GIC) liner (Vitrebond, 3M ESPE, St. Paul, MN, USA; or Fuji VII pink, GC corp., Tokyo, Japan) was placed as an orifice barrier. The cavity was bonded with an etch-and-rinse (Single Bond 2, 3M ESPE) or self-etching (Clearfil SE Bond, Kuraray, Osaka, Japan) adhesive. The remainder of the cavity was filled with a nano-hybrid or micro-hybrid resin composite (Filtek Z250 or Z350, 3M ESPE), which was light-cured for 20-40 sec for each layer. The centric and eccentric occlusal relationships were evaluated and adjusted before the restoration was finished and polished.

For the premolars restored with crowns, a fibre-reinforced post (e.g. DT Light Post, Bisco Inc., Schaumburg, IL, USA) was tried and cemented into the prepared post space, with at least 4 mm of the gutta-percha remaining. The prefabricated post was cemented with a resin-based core build-up material (e.g. MultiCore Flow, Ivoclar Vivadent AG, Schaan, Liechtenstein). Then, either an all-ceramic or porcelain-fused-to-metal crown was fabricated and cemented using a self-adhesive resin cement (RelyX Unicem or RelyX U200, 3M ESPE).

### Outcome assessment

The occurance of tooth fracture was recorded. The primary outcomes were fractured or survived without fracture. Next, the fracture location was identified as a coronal, coronal-root, or root fracture. The fractures were finally categorised as restorable (repaired or replaced with a new restoration) or non-restorable (required tooth extraction).

### Statistical analysis

The data were statistically analysed using the Statistical Package for the Social Sciences (SPSS) version 22.0 (SPSS Inc., Chicago, IL, USA) with statistical significance set at P<0.05. The survival rates against fracture were compared between the teeth restored with crowns and those restored with resin composites with a log-rank test. The survival periods without fracture were analysed by a Kaplan-Meier survival analysis. Cox proportional hazard regression analysis was used to determine the association between the incidence of fracture and the prognostic factors with P≤0.20 in the univariate analysis.

## RESULTS

The endodontically treated teeth comprised 87 premolars from 77 patients (20 males and 57 females) with ages between 24-87 years (mean 58.2±11.6 years) were included. In this study, 10 patients had 2 ETT, and the remainder (67) had one ETT. The teeth had exposed cervical lesions on the buccal side. Among the 87 ETT, 30 teeth were restored with full-coverage crowns, and 57 teeth were restored with resin composites. The follow-up periods were 37.5±15.0 months (14-60 months) for the teeth with crowns and 29.7±16.2 months (12-60 months) for the teeth with resin composite. The distribution of the factors based on the coronal restorations is presented in Table 1.

**TABLE 1.** Data distribution of the endodontically treated premolars with exposed cervical lesions that were restored with crowns or resin composites

Factors	Crown (n=30)		Resin composite (n=57)		Total (n=87)	
	n	%	n	%	n	%
Sex						
Male	8	26.7	16	28.1	24	27.6
Female	22	73.3	41	71.9	63	72.4
Age (years old)						
Less than 50	5	16.7	7	12.3	12	13.8
≥50	25	83.3	50	87.7	75	86.2
Tooth location						
Maxillary teeth	8	26.7	18	31.6	26	29.9
Mandibular teeth	22	73.3	39	68.4	61	70.1
Level of cervical lesion						
Cervical third	9	30.0	12	21.1	21	24.1
Middle third	21	70.0	45	78.9	66	75.9
Opposing tooth						
Natural tooth	28	93.3	57	100	85	97.7
Fixed restoration	2	6.7	-	-	2	2.3
Proximal contact						
2 sides	20	66.7	33	57.9	53	60.9
0-1 side	10	33.3	24	42.1	34	39.1
Posterior support						
Bilateral	28	93.3	54	94.7	82	94.3
Unilateral or none	2	6.7	3	5.3	5	5.7
Abutment function						
No	28	93.3	56	98.2	84	96.6
Yes	2	6.7	1	1.8	3	3.4
Parafunctional habit						
No	28	93.3	53	93.0	81	93.1
Yes	2	6.7	4	7.0	6	6.9
Crestal bone level						
Coronal	29	96.7	46	80.7	75	86.2
Middle	1	3.3	11	19.3	12	13.8

There was no significant difference in the data distribution between the two groups (Fisher's exact test; P≥0.05)

The overall survival rate of the premolars with exposed cervical lesions was 88.5% (77/87 teeth). The survival rate in the teeth with a crown was 93.3% (28/30 teeth), and 86% (49/57 teeth) for those restored with resin composite. The survival rates based on each factor are shown in Table 2.

**TABLE 2.** Univariate analysis of the survival rates against fracture in endodontically treated premolars with exposed cervical lesions (n=87)

Factors	Number of teeth	Survived without fracture n (%)	Fractured n (%)	P
Restoration type				
Crown	30	28 (93.3)	2 (6.7)	0.20*
Resin composite	57	49 (86.0)	8 (14.0)	
Sex				
Male	24	22 (91.7)	2 (8.3)	0.44
Female	63	55 (87.3)	8 (12.7)	
Age (years old)				
Less than 50	12	10 (83.3)	2 (16.7)	0.21
≥50	75	67 (89.3)	8 (10.7)	
Tooth location				
Maxillary	26	23 (88.5)	3 (11.5)	0.47
Mandibular	61	54 (88.5)	7 (11.5)	
Level of cervical lesion				
Cervical third	21	20 (95.2)	1 (4.8)	0.35
Middle third	66	57 (86.4)	9 (13.6)	
Proximal contact				
2 sides	53	47 (88.7)	6 (11.3)	0.48
0-1 side	34	30 (88.2)	4 (11.8)	
Parafunctional habit				
No	81	72 (88.9)	9 (11.7)	0.16*
Yes	6	5 (83.3)	1 (16.7)	
Crestal bone level				
Coronal	75	68 (90.7)	7 (9.3)	0.01*
Middle	12	9 (75.0)	3 (25.0)	

*The univariable analysis revealed the three potential predisposing factors that were further included in the Cox proportional hazards model multivariate analysis: Restoration type, parafunctional habit, and crestal bone level (P≤0.20)

**Figure 1.**
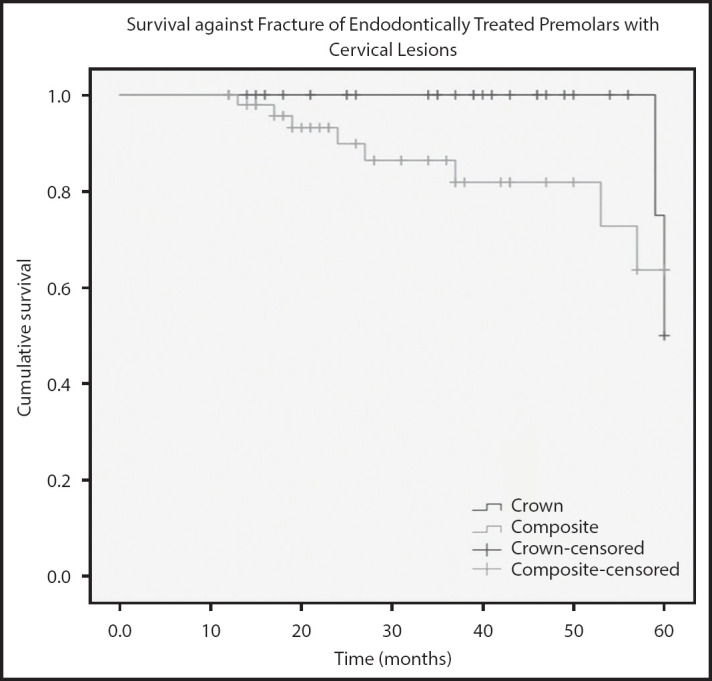
The Kaplan-Meier cumulative survival curves of endodontically treated premolars with exposed cervical lesions restored with crowns or resin composites. The log-rank analysis did not show a significant difference in the survival rates against fracture between the premolars restored with either of the two types of coronal restoration (P≥0.05)

The Kaplan-Meier cumulative survival curves (Fig. 1) demonstrated that the incidence of fracture in the teeth with resin composite restorations, beginning at the first year, was earlier than in those with crowns. From the log-rank analysis, the survival rates of the premolars restored with crowns or resin composites were not significantly different (P≥0.05).

The fractures found in the 2 teeth with crowns were a crown-root fracture and a vertical root fracture. Out of the 8 fractured teeth with resin composite restorations, 6 teeth had crown-root fractures and 2 teeth had vertical root fractures. All fractures (100%) in the premolars with crowns were non-restorable, whereas, 87.5% of the fractured premolars with resin composites were non-restorable. 

The univariable analysis revealed three potential prognostic factors that were further included in the multivariate analysis (Table 2), which were restoration type, parafunctional habits, and crestal bone level, with P≤0.20. The Cox proportional hazards model (Table 3) indicated that the crestal bone level was the significant prognostic factor for the survival rate against the fracture of endodontically treated premolars with exposed cervical lesions (P<0.05). The teeth with the crestal bone level reduced to the middle-third of the root had a 5.85-fold higher chance of fracture compared with those with the crestal bone in the coronal-third of the root (hazard ratio=5.85; 95% confidence interval, 1.11-30.70; P<0.05).

**TABLE 3.** Cox proportional hazards model of the three potential predisposing factors for survival against fracture in endodontically treated premolars with exposed cervical lesions

Factors	Hazard ratio (95% CI)	P
Restoration type		
Resin composite vs. Crown	2.07 (0.42-10.26)	0.37
Parafunctional habit		
Yes vs. No	7.84 (0.77-79.75)	0.08
Crestal bone level		
Middle vs. Coronal	5.85 (1.11-30.70)	0.04*

*A significant difference was indicated by the Cox regression analysis (P<0.05). CI: Confidence interval

## DISCUSSION

The present study compared the survival rates against fracture of endodontically treated premolars with exposed cervical lesions that were restored with either crowns or resin composites, and determined the prognostic factors for fracture. Within the 5-year observation period, the survival rates against fracture of the endodontically treated premolars with exposed cervical lesions were high and comparable between the teeth restored with crowns (93.3%) and resin composites (86%). To the best of our knowledge, this is the first clinical study to investigate the survival rate against the fracture of ETT with exposed cervical lesions. Our results suggested that the endodontically treated premolars could be conservatively restored with resin composite as the permanent restoration (7); the placement of a full-coverage crown is not mandatory.

Although similar survival rates were observed for the two restoration types, there are no prior clincial studies to compare our results with. However, our clinical findings corresponded with the results of a laboratory study, wherein comparable fracture resistance was observed between the endodontically treated premolars with exposed cervical lesions restored with all-ceramic crowns and resin composites (11).

A cervical lesion generates high-stress that concentrates on the cervical region and changes the stress-strain pattern, which reduces the fracture resistance of ETT (8-13). However, this adverse effect can be reversed by restoring the cavity with a resin composite, which reverses the biomechanical behaviour of an ETT to the level of an intact tooth (8, 10-13). Therefore, the survival rate against fracture of the ETT restored with resin composites was comparable with those of crowns. Additionally, the conservative approach when using a direct restoration is beneficial in preserving the natural tooth structure, reducing chair time, and increasing cost-effectiveness.

More than half of the cases in this study had two adjacent teeth and no parafunctional habits, these conditions were favourable for the survival from fracture of the evaluated teeth. The proximal contacts of the adjacent teeth improve the survival rate against the fracture of ETT (7, 14), by distributing occlusal forces and reducing functional loading on the teeth. Many clinical studies reported the benefit of existing proximal contacts or adjacent teeth on ETT (7, 14, 15). With two proximal contacts, endodontically treated premolars with a moderate loss of tooth structure were successfully restored with resin composites, achieving a high survival rate comparable to that with crowns (7). In the absence of parafunctional habits, the occlusal forces are limited, which tends to decrease the chance of postoperative fracture in ETT.

Most patients included in this study were females, and a higher precentage of the females attended the follow-up compared with males. This studied population may be a confounding factor to the outcome because the occlusal force in females tends to be lower compared with males (16). However, no significant difference in the survival without fracture was found between the two sexes in this study.

In our study, teeth with root canals prepared by hand and/or rotary files using the crown-down technique were included. The instrumentation provided a final taper between 4-6% and did not excessively remove root dentin or weaken the root structure. In addition, the different obturation methods, lateral and warm vertical compaction, may not influence whether a root-filled tooth fractures if the obturation was performed appropriately.

The protocol for restoring ETT with resin composite was specific. All the teeth received a radicular extension of the resin composite, into approximately the coronal-third of the root canal, after 1-3 mm of the root canal filling below the canal orifice was removed. The primary goal of this protocol was to create an internal seal at the cervical exposure site to prevent coronal leakage into the filled root canal if any dislodgement of the cervical restoration occured. Furthermore, the radicular extension of the bonded restoration may improve the fracture strength of ETT by internal reinforcement, thereby reducing the chance of cervical fracture (17, 18). Therefore, this specific restorative protocol enhanced the survival from fracture of endodontically treated premolars with an exposed cervical cavity in our study.

A crown-root fracture was most commonly found in the premolars with exposed cervical lesions. All fractures occurred at the cervical region where the lesion was located, leaving only a retained root. The restorability of the fractured ETT is then reduced, because the remaining tooth structure and its ferrule effect were limited. The absence of a ferrule compromises the prognosis and may lead to extraction of the fractured tooth; thus, it is critical for the longevity of ETT (19, 20). If the cervical restoration degrades, it must be replaced to maintain support for the tooth and to decrease the chance of a cervical fracture (10, 21).

In this study, a post was rarely placed in the cases restored with resin composite, while the crowned teeth were restored with a post. Most of the posts used in this study were prefabricated fibre posts that may provide a lower risk of non-restorable fracture compared with using a rigid cast post. However, the preparation of a post space tends to weaken the remaining root structure by further enlargement of the root canal. The fracture incidence of the crowned teeth with posts in our study was 6.7%.

The crestal bone level was the significant factor for survival against fracture. Compared with the normal crestal bone level at the cervical-third, the endodontically treated premolars with the bone support reduced to the middle-third of the root had a greater risk of tooth fracture. Previous clinical studies have reported the negative effect of the loss of bone support on the long-term survival of teeth (22, 23). The fracture resistance of ETT is affected by the level of bone support, and horizontal bone loss increases the risk of fracture *in vitro* (24). This is consistent with our clinical findings in which the crestal bone level was a significant prognostic factor for the survival from fracture of the premolars.

## CONCLUSION

For endodontically treated premolars with exposed cervical lesions, direct resin composite restorations with a radicular extension below the exposure site provided a high survival rate against fracture, comparable to that of crowns. Crestal bone loss to the middle-third of the root was a significant prognostic factor for tooth fracture.
